# Premature stroke and cardiovascular risk in primary Sjögren's syndrome

**DOI:** 10.3389/fcvm.2022.1048684

**Published:** 2022-12-14

**Authors:** Clara L. Zippel, Sonja Beider, Emelie Kramer, Franz F. Konen, Tabea Seeliger, Thomas Skripuletz, Stefanie Hirsch, Alexandra Jablonka, Torsten Witte, Kristina Sonnenschein, Diana Ernst

**Affiliations:** ^1^Department of Rheumatology and Immunology, Hannover Medical School, Hannover, Germany; ^2^Department of Neurology, Hannover Medical School, Hannover, Germany; ^3^Department of Cardiology, Hannover Medical School, Hannover, Germany

**Keywords:** Sjögren syndrome, Raynaud disease, atherosclerosis, cardiovascular risk factors, cardiovasclar disease, vasculitis, EULAR Sjögren's Syndrome Disease Activity Index (ESSDAI), stroke

## Abstract

**Introduction:**

Primary Sjögren's syndrome (pSS) is associated with an increased prevalence of traditional risk factors and cardiovascular diseases (CVDs). The study aimed to identify specific risk factors for CVD in pSS patients.

**Methods:**

PSS patients with and without CVD were compared. All patients fulfilled the EULAR/ACR classification criteria. Patients with CVD presented at least one of the following manifestations: myocardial infarction, transient ischemic attacks, ischemic or hemorrhagic stroke, peripheral artery disease, coronary artery disease, and carotid plaques. Data were collected by a standardized protocol and review of medical records.

**Results:**

61/312 (19.6%) pSS patients presented with CVD. Traditional risk factors such as hypertension, hypercholesterinemia and diabetes (*p* < 0.05), pSS manifestations, in particular vasculitis (*p* = 0.033) and Raynaud's phenomenon (*p* = 0.018) were associated with CVD. Among patients with ischemic events (28/312, 9%), particularly cerebrovascular disease (*n* = 12/28, 42.9%), correlations with increased EULAR Sjögren's Syndrome Disease Activity Index (ESSDAI) (*p* = 0.039) and EULAR Sjögren's Syndrome Patient Reported Index (ESSPRI) (*p* = 0.048) were observed. Age at first cerebrovascular event was 55.2 [48.9–69.6] years. Multivariate analysis confirmed hypertension [odds ratio (OR) 3.7, 95% confidence interval (CI) 1.87–7.18, *p* < 0.001], hypercholesterinemia (OR 3.1, 95% CI 1.63–5.72, *p* < 0.001), male gender (OR 0.4, 95% CI 0.17–0.78, *p* = 0.009), Raynaud's phenomenon (OR 2.5, 95% CI 1.28–4.82, *p* = 0.007), and CNS involvement (OR 2.7, 95% CI 1.00–7.15, *p* = 0.048) as independent CVD predictors.

**Conclusion:**

Raynaud's phenomen as well as vasculitis and high ESSDAI have shown a significant association to CVD. PSS patients with cerebrovascular events were younger than expected. Knowledge about risk factors may help clinicians to identify pSS patients at risk for CVD. After diagnosis of pSS, patients should be screened for risk factors such as hypertension and receive appropriate therapy to prevent or at least reduce sequelae such as infarction. However, further investigations are necessary in order to achieve a reliable risk stratification for these patients.

## Introduction

Primary Sjögren syndrome (pSS) is a common connective tissue disease with an estimated prevalence of 1:100 to 1:1,000, predominantly occurring in middle-aged females (10:1) (1). In general, pSS is characterized by lymphocytic infiltration of exocrine glands, typically resulting in ocular and oral dryness. Manifestations can however be diverse, with almost 50% of patients displaying extra-glandular manifestations (2). This appears mainly driven by systemic inflammation of various end-organs, captured within the EULAR Sjögren's Syndrome Disease Activity Index (ESSDAI) (3). Recent data revealed that more than a quarter of patients may have manifestations currently not included in the ESSDAI, with cardiovascular (CV) involvement being the most frequent (4).

CVD risk is known to be independently increased in both systemic lupus erythematosus and rheumatoid arthritis (5), and given the immunological similarities to pSS, it remains an area of interest in this cohort. It has previously been shown, that increased rates of atherosclerosis are evident in pSS populations (6). While exact mechanisms remain unknown, various studies have identified endothelial dysfunction, intimal thickening and resulting loss in vessel wall compliance as being evident in pSS cohorts (7).

PSS does however not provide immunity from traditional CVD risk, with recent epidemiological data actually correlating pSS with traditional risk factors, especially hypertension and hypercholesterinemia (8–10). One exception is smoking, with several studies suggesting lower prevalence, likely due to oral discomfort (8, 9). The results for the remaining risk factors are inconclusive. For example, two studies observed a higher frequency of diabetes mellitus (11, 12) while Bartoloni et al. (8) presented opposing results. Nevertheless, a current meta-analysis confirmed a subsequently increased risk for cardiovascular and cerebrovascular events (13).

Aim of this retrospective study is to analyze differences between pSS patients with and without CV involvement, focusing on traditional risk factors, immunological parameters, comorbidities, and extra-glandular pSS manifestations.

## Materials and methods

### Study population

We retrospectively analyzed clinical data of all confirmed pSS patients attending the outpatient clinics of either the Rheumatology or Neurology Departments at Hannover Medical School between January 2018 and March 2021. Patients were classified according to either the 2016 American College of Rheumatology/European League Against Rheumatism Classification Criteria (14) or the 2012 American College of Rheumatology classification criteria (15) depending on time of diagnosis.

### pSS diagnosis and evaluation

Clinical data were collected using previously performed, standardized questionnaires. This included age at diagnosis, current symptom profiles and evidence of extra-glandular or systemic involvement based upon ESSDAI, serological tests including antinuclear antibodies, rheumatoid factor, alpha-fodrin, anti-SSA/Ro and anti-SSB/La antibodies, along with differential blood counts and standard biochemistry. Disease activity was scored using ESSDAI (3) and severity of symptoms by EULAR Sjögren's Syndrome Patient Reported Index (ESSPRI) (16). Xerostomia and xerophthalmia were confirmed by a positive Saxon test [ < 3.5 g in 2 min (stimulated saliva flow)] and/or positive Schirmer test [ < 5 mm in 5 min (lacrimal flow)]. Sonographic assessment of salivary gland involvement was performed and scored according to the DeVita system (17). When indicated, minor salivary gland biopsy was obtained [scored according to Chisholm and Mason (18)]. The study protocol was approved by the local Ethics committee (8179_BO_S_2018) and declaration of consent was received from all the subjects.

### Cardiovascular assessment

Traditional CVD risk factors, manifest CVD events, and objective evidence of asymptomatic atherosclerotic disease were assessed and recorded using standardized criteria, applied when reviewing patient medical records. Traditional CVD risk factors were defined as follows: hypertension (diagnosis by a cardiologist and/or ongoing antihypertensive therapy), hypercholesterinemia (physicians' diagnosis and/or use of lipid-lowering agents), smoking (previous/current or never smoking, pack years), diabetes mellitus (physicians' diagnosis and/or ongoing therapy with oral hypoglycemic agent or insulin), obesity (based on body mass index), and positive family history (CVD event in a first-degree relative ≥65 yrs). Further CV comorbidities like heart failure and atrial fibrillation were assessed. Finally, CVD was recorded as proven myocardial infarct (MI) or catheter-proven coronary artery disease, proven cerebrovascular disease including transient ischemic attacks (TIA) or known carotid artery disease on Duplex sonography and peripheral vascular disease (PVD). PVD was diagnosed by clinical symptoms and duplex sonography findings. All patients in our cohort diagnosed with PVD were symptomatic (Fontaine classification ≥2) (19). CV events only included MI, stroke and proven PVD.

### Statistical analysis

Quantitative variables are expressed as mean and SD, whereas categorical variables are reported as frequency (*n*) and percentage (%). The variables were assured with Pearson's correlation and subsequently with partial correlation, therefore age was excluded as a confounding variable. According to Kolmogorov–Smirnoff, the Gaussian distribution was controlled. Parametric and Non-parametric tests were applied to verify the distribution of each risk factor within the investigated groups. Significant results were proved according to the Chi square test, Mann–Whitney-*U*- and Kruskal–Wallis-tests. Furthermore, the median values were compared by using the Hodges–Lehmann-test, where the two-sided confidence interval of 95% was given. For categorical variables, missing values have been excluded. The Bonferroni correction to neutralize the type 1 error by multiple statistical tests was performed. Finally, we conducted a multivariate analysis to determine variables that were independently associated with CVDs. A two-sided significance level was set at 0.05 for all test procedures. The statistical data analysis was obtained by using SPSS software (SPSS 27, IBM Corporation and its licensors 1989, 2020).

## Results

### Characteristics of the study population

In total 312 patients were included in the cohort. Of these, 251/312 (80.4%) were female. Age at inclusion was 58.3 ± 14 years and mean disease duration was 5.0 ± 6.7 years. Two hundred forty-seven pSS patients were classified according to EULAR/ACR 2016 and 65 patients according to the 2012 ACR classification criteria (14, 15).

More than two-thirds of patients (67.9%) exhibited objective xerophthalmia and 168 (53.8%) patients showed an objective xerostomia. A total of 181 (58.0%) of the cohort underwent salivary gland biopsy and 146 (80.7%) of them had a Chisholm and Mason score ≥3. In addition, we performed ultrasounds of the salivary glands in 311 patients, 96 (30.9%) of these presented a DeVita score ≥ 4. Few patients were detected with scleroderma associated antibodies (*n* = 21), all of them presenting Raynauds phenomen, but the number in the CVD and control group did not differ significantly (Table 1).

**Table 1:** General characteristics of the study population (*n* = 312).

**Characteristic**	***n* (%)**
Age, mean ± SD; median (min–max)	58.3 ± 14; 59 (21–87)
Gender (male)	61 (19.6%)
Xerophthalmia	212 (67.9%)
Xerostomia	168 (53.8%)
Salivary gland biopsy	181 (58.0%)
Salivary gland biopsy focus score ≥1	146/181 (80.7%)
**Extra-glandular and systemic manifestations**	
Depression	87 (27.9%)
Fatigue	236 (75.6%)
Myalgia	160 (51.3%)
Raynaud's phenomenon	108 (34.6%)
Articular involvement	213 (68.3%)
Lung involvement	48 (15.4%)
PNS involvement	107 (34.3%)
CNS involvement	29 (9.3%)
Liver involvement	6 (1.9%)
Renal involvement	6 (1.9%)
Cutaneous involvement	19 (6.1%)
Fever	7 (2.2%)
Night sweats	40 (12.8%)
Weight loss	18 (5.8%)
**Laboratory measurements**	
Antinuclear antibodies (>1:160)	226 (72.4%)
Alpha-fodrin antibodies (>15 U/ml)	115 (36.9%)
Rheumatoid factor (>14 IU/ml)	91 (29.2%)
Anti-SSA/Ro antibodies (>7 U/ml)	166 (53.2%)
Anti-SSB/La antibodies (>7 U/ml)	59 (18.9%)
Hypergammaglobulinaemia (>16 g/l)	72 (23.1%)
**Treatment**	
Glucocorticoids	153 (49.0%)
Hydroxychloroquine	177 (56.7%)
Immunosuppressants	170 (54.4%)

IU, international units; Anti-SSA, Anti-Sjögren's-syndrome-related antigen A autoantibodies; Anti-SSB, Anti-Sjögren's-syndrome-related antigen B autoantibodies.

Tables 1, 2 summarizes the general characteristics of our pSS cohort.

**Table 2:** Comparison between pSS patients with and without cardiovascular and atherosclerotic endpoints.

**Characteristics**	**Index patients** **(*n* = 61)**	**Controls** **(*n* = 251)**	***P*-value**
Age, mean ± SD, median (min–max)	64.7 ± 12.2, 66 (30–85)	56.7 ± 14.1, 58 (21–87)	**<0.001**
Gender (male)	18 (29.5%)	43 (17.1%)	**0.029**
Xerophthalmia	44 (72.1%)	168 (66.9%)	0.506
Xerostomia	31 (52.5%)	137 (55.2%)	0.708
Salivary gland biopsy	36 (59%)	145 (57.8%)	
Salivary gland biopsy focus score ≥1	31/36 (86.1%)	114/145 (78.6%)	0.448
Disease duration	4.7 ± 6.1	5.0 ± 6.7	0.543
Hypertension	45 (73.8%)	96 (38.2%)	**<0.001**
Hypercholesterinemia	34 (55.7%)	60 (23.9%)	**<0.001**
Diabetes mellitus	14 (23.0%)	28 (11.2%)	**0.016**
Smoking^*^	36 (59.0%)	117 (46.6%)	0.067
BMI, mean ± SD	26.5 ± 5.1	26.9 ± 5.9	0.657
Physical activity	30 (49.2%)	152 (60.6%)	0.131
Family history	25 (41.0%)	109 (43.4%)	0.730
Thrombosis	10 (16.4%)	32 (12.7%)	0.455
AI-thyreoditis	10 (16.4%)	42 (16.7%)	0.949
Heart failure	13 (21.3%)	11 (4.4%)	**<0.001**
Atrial fibrillation	10 (16.4%)	12 (4.8%)	**0.002**
Antinuclear antibodies	47 (77.0%)	179 (71.3%)	0.370
Rheumatoid factor	16 (26.2%)	75 (29.9%)	0.580
C3 > 1.8 g/l	0(0)	5 (2%)	0.539
C4 > 0.4 g/l	0 (0)	4 (1.6)	0.303
IgG4 > 1.4 g/l	5 (8.2%)	11(4.4%)	0.829
Scl70	0 (0%)	1 (0.4%)	0.543
CenpB	3 (4.9%)	7(2.8%)	0.647
U1RNP	3 (4.9%)	7 (2.8%)	0.456
Cardiolipin Ak	2 (3.3%)	2 (0.8%)	0.122
Alpha-fodrin antibodies	25 (41.0%)	90 (35.9%)	0.515
Anti-SSA/Ro antibodies	30 (49.2%)	136 (54.4%)	0.859
Anti-SSB/La antibodies	8 (13.1%)	51 (20.3%)	0.497
Hypergammaglobulinaemia	13 (21.3%)	59 (23.5%)	**0.024**
ESR, mean ± SD	18.4 ± 16.7	19.2 ± 19.3	0.621
CRP, mean ± SD	3.6 ± 5.7	3.8 ± 7.0	0.999
Vasculitis	7 (11.5%)	11 (4.4%)	**0.033**
Raynaud's phenomenon	29 (47.5%)	79 (31.5%)	**0.018**
Lung involvement	14 (23.0%)	34 (13.5%)	0.068
Peripheral neuropathy	28 (45.9%)	79 (31.5%)	**0.034**
ESSDAI, mean ± SD	12.2 ± 8.8	10.2 ± 9.2	0.061
ESSPRI, mean ± SD	4.8 ± 3.0	4.3 ± 3.0	0.315
HCQ	31 (50.8%)	146 (58.2%)	0.300
GCC	28 (45.9%)	125 (49.8%)	0.585

SD, standard deviation; BMI, Body-Mass-Index; Anti-SSA, anti–Sjögren's-syndrome-related antigen A autoantibodies; Anti-SSB, anti–Sjögren's-syndrome-related antigen B autoantibodies; ESR, erythrocyte sedimentation rate; CRP, C-reactive protein; ESSDAI, EULAR Sjögren's syndrome disease activity index; ESSPRI, EULAR Sjogren's Syndrome Patient Reported Index; HCQ, Hydroxychloroquine; GCC, Glucocorticoids; IS, Immunosuppressants.

* Smoking compares ever and current smokers against never smokers.

Bold values indicate the significant values.

### Comparison between pSS patients with and without cardiovascular and atherosclerotic endpoints

In total 61/312 (19.6%) patients were identified as having some form of CVD, with the remainder acting as comparative controls (Table 2). CVD patients tended to be older (64.7 ± 12.2, median 66 (30–85) years, *p* < 0.001) and a greater proportion were male (29.5 vs. 17.1%; *p* = 0.029). No difference in pSS duration was evident between groups. Established CVD risk factors such as hypertension (73.8 vs. 38.2; *p* < 0.001), hypercholesterinemia (55.7 vs. 23.9%; *p* < 0.001) and diabetes mellitus (23.0 vs. 11.2%; *p* = 0.016) were more prevalent in CVD patients. Smoking prevalence was higher in CVD patients (59.0 vs. 46.6%; *p* = 0.067), the difference was not significant. Neither family history of CVD nor BMI differed between groups (both *p* > 0.05). Non surprisingly CVD patients had more CVD-associated comorbidities such as atrial fibrillation (16.4 vs. 4.8%; *p* = 0.002) and heart failure (21.3 vs. 4.4%; *p* < 0.001).

CRP, ESR, complement, and autoantibodies were comparable in both groups. CVD patients with manifest atherosclerosis exhibited higher rates for vasculitis (11.5 vs. 4.4%; *p* = 0.033) and Raynaud's phenomenon (47.5 vs. 31.5%; *p* = 0.018). Neither glucocorticoid (GC) nor immunosuppressive therapy demonstrated any significant difference between these two groups. There was no association of GCs and hypertension, diabetes mellitus, or hypercholesterinemia in this cohort.

### Comparison between pSS patients with and without overt ischemic events

At least one ischemic event, MI, stroke, or PAD, was confirmed in 28/312 (9%) patients (Supplementary Table 1). The remaining (*n* = 284) were defined as control. As expected, results regarding modifiable and non-modifiable risk factors remained mostly constant. Although absolute numbers are small in addition to the prior results, CNS involvement now correlated with overt ischemic events [6/28 (21.4%) vs. 23/284 (8.1%), *p* = 0.021].

Age at first cerebrovascular event was 55.2 [48.9–69.6] years. Cerebrovascular events were not associated to arterial fibrillation.

Compared to the control, patients with at least one ischemic manifestation had a higher disease activity (ESSDAI score ≥ 5) (*p* = 0.039). Consequently, the mean ESSDAI score in these patients was 13.5 ± 7.7 (*p* = 0.021). With regard to ESSPRI, we found a similar tendency, a high ESSPRI (*p* = 0.048) was associated with ischemic events.

Despite small numbers, we searched for noticeable differences between pSS patients with manifested MI (*n* = 8), stroke (*n* = 12), or PAD (*n* = 8). All documented strokes were of ischaemic nature. Of interest, anti-SSB/La positivity was more prevalent in pSS patients with MI (*p* = 0.017), whereas cerebrovascular events were significantly associated with a high CNS domain of the ESSDAI (*p* = 0.021). Patients with PAD were less likely to be treated with HCQ (*p* = 0.005).

### Predictors of CVDs

Finally, we performed a multivariate analysis, which revealed hypertension, hypercholesterinemia, male gender, Raynaud's phenomenon, and CNS involvement as independent predictors of CVDs in patients with pSS (*p* < 0.05; Table 3).

**Table 3:** Predictors of cardiovascular disease, multivariate analysis of gender, Raynaud's phenomenon, hypertension, hypercholesterolemia, CNS involvement (OR Odds Ratio, CNS central nerve system).

	**OR**	**95% CI**		***P*-value**
		**Lower**	**Upper**	
Gender (male)	0.363	0.170	0.775	0.009
Raynaud's phenomenon	2.484	1.281	4.818	0.007
Hypertension	3.659	1.865	7.181	< 0.001
Hypercholesterinaemia	3.051	1.628	5.717	< 0.001
CNS involvement	2.684	1.007	7.153	0.048

## Discussion

This is the first study to our knowledge reporting about premature stroke in Sjögren Syndrome and Raynaud's phenomen as a potential risk factor for CVD in patients with pSS. Whilst vasculitis in various diseases is known to be able to involve coronary arteries, there is no previous data for pSS patients with Raynaud's phenomenon suffering from more CVD (20–22). Although several studies clearly demonstrated, that pSS patients have an increased prevalence of CV risk factors and overt CVDs (8, 13).

In the present study, almost one-fifth of pSS patients were affected by CV involvement. Comparing only presence of MI, other coronary heart disease, heart failure, or stroke in a normal German female age matched cohort you would expect 8–16% suffering of the mentioned diseases. Although these cohorts are not matched, the prevalence of those diseases seems much higher in patients with pSS (23). Next to known cardiac risk factors (cRF), disease-specific manifestations, such as vasculitis and Raynaud's phenomenon were also more common in pSS patients with CVD, suggesting a possible synergistic effect between traditional risk factors and disease-specific factors resulting in a higher atherogenic potential and increased CV burden.

Interestingly in our cohort, patients with stroke were remarkably younger at first cerebral event than expected in an average German cohort (24). As we could not see an association to atrial fibrillation or other obvious risk factors an association of pSS is suggestive.

Due to the inclusion of patients with neurological involvement of Sjögren's syndrome, the proportion of male gender in our cohort is larger than expected. In neurological cohorts, the gender proportion is balanced, unlike in rheumatological cohorts [61/312 (19.6%) were male] (25). In line with other studies, male gender and older age were significantly associated with CVDs (8, 11, 12). On the contrary, disease duration had no impact in our cohort, despite the fact that recent studies were able to establish an association between disease duration and CV events (8, 26). However, our results may be explained by the heterogenous course, some late presenters and the possibility of CVD to occur as one of the first symptoms in patients with pSS (27, 28).

It is undeniable that traditional risk factors have the greatest impact in the development of atherosclerotic manifestations and CV burden (29). In case of pSS, our multivariate analysis confirmed that hypertension and hypercholesterolemia play a pivotal role as predictors of CVDs. Among other classical risk factors, DM was more frequent in pSS patients with CV events, whereas sedentary lifestyle and BMI were similarly distributed between index patients and control. Our results agree with Cai et al. (11), who observed a higher frequency of hypertension, hypercholesterinemia, DM plus an increased BMI in pSS patients with CV involvement. In addition, Bartoloni et al. (26) recently demonstrated a strong association between CV risk factors and CVDs in pSS patients, with hypertension playing a central role. The increased prevalence of risk factors leading to increasing CV events, demands improvement in the prevention and management of pSS. In line with this a recent study showed a far higher prevalence for subclinical atherosclerosis in pSS compared to an age and sex matched control group (30). The authors report an association of an elevated erxthrocyte sedimentation rate and rheumatoid factor with subclinical atherosclerosis in pSS patients. A review from Bartoloni et al. reports about the role of inflammation in the pathogenesis of pSS (31) supporting findings that patients with a higher ESSDAI and presence of SSA antibodies present more often endothelial dysfunction (7, 32).

Of note, despite adjusting for traditional risk factors, Wu et al. (33) found an increased hazard ratio of 1.52 in pSS patients with coronary heart disease, suggesting that the disease itself is a risk factor. Therefore, we investigated in a wide range of disease-specific laboratory and clinical markers. Our results are similar to findings of Italian studies, that demonstrated an increased frequency of immunological abnormalities in pSS patients without traditional risk factors and failed to establish a direct association between autoantibodies and CV events (8, 26). However, a similar amount of data strengthens the opposing hypothesis that immunological and inflammatory abnormalities correlate with atherosclerotic manifestations and thereby also with CVDs. For instance, Mofors et al. (34) have shown a higher risk of cerebral infarction and venous thromboembolism in pSS patients positive for anti-SSA/Ro and anti-SSB/La antibodies. We saw a higher prevalence of anti-SSB/La positivity in pSS patients with MI (*p* = 0.017), whilst we did not see an association with venous thromboembolism. But numbers are small and therefore these results have to be interpretated with caution.

Interestingly we found a slightly lower presence of hypergammglobulinemia in patients with CVD, similar findings have been shown before by Pérez-De-Lis et al. in a pSS cohort of the same size as ours (*n* = 312) (12). They detected in the multivariate analysis for patients with more than three cRF a higher frequency of CNS involvement, which fits to our results as well.

Clinical markers including vasculitis and Raynaud's phenomenon can play a crucial role in relation to CVDs. Based on our subanalysis, we identified night sweats as an additional association with ischemic events (Supplementary Table 1). As night sweats can be an indicator for disease activity (3) or systemic inflammation, this finding is easily explained as both have been shown to increase CVD risk (35). On the other hand night sweats can be caused by many other reasons like menopause and autonomic neuropathy (36). Especially early menopause and autonomic neuropathy may increase cardiovascular risk in pSS patients as well (37, 38).

Through multivariate analysis we confirmed that Raynaud's phenomenon is independently associated with CVDs. A correlation between Raynaud's phenomenon and autonomic neuropathy (39) as well as microvascular peripheral endothelial dysfunction, one of the early stages of atherosclerosis, has been shown before (40). Besides microvascular changes, even a correlation between subclinical myocardial fibrosis and Raynaud's phenomenon was discovered, giving additional support for our results, showing Raynaud's phenomenon is an independent predictor (41). Both, Raynaud's phenomenon and vasculitis, are among the most common extra-glandular manifestations in pSS patients, whereas vasculitis is particularly associated with increased mortality (42). Of consequence, special precautions like frequent CV monitoring and tight control of CV risk factors like arterial hypertension, diabetes mellitus, and hyperlipidemia are indispensable. That is why it is so important to recognize and use the EULAR guidelines for the management of cardiovascular comorbidities (43).

A characteristic feature of patients with CVDs was a more pronounced systemic manifestations than glandular involvement. Among the systemic manifestation, polyneuropathy was the most common one, in part due to the inclusion of patients with neurologic involvement from the Department of Neurology. Additionally, our subanalysis revealed CNS involvement as a significant factor for ischemic events, which was also confirmed by the multivariate analysis. Considering that cerebral vasculitis frequently results in stroke, these findings are not surprising (44). Similar results regarding the influence of extra-glandular manifestations on CV events were obtained by studies from China (11) and Italy (8).

In our study, ischemic events were associated with a high to moderate ESSDAI and a high ESSPRI. A closer look at the individual domains of the ESSDAI score revealed four possible trends (constitutional, renal, PNS, CNS), which may be significant in a larger cohort. Agreeing with our finding, lupus patients with carotid intima media thickness and carotid plaques were associated with a severe inflammatory status expressed by the systemic lupus erythematosus disease activity index (SLEDAI) (45), a counterpart of the ESSDAI.

Due to the higher risk of CVD in patients with rheumatic diseases the EULAR recommends an adaption of CVD risk calculators by a 1.5 multiplication factor in patients with rheumatoid arthritis (43). Not only the implementation of altered risk models, also a targeted CV management with routine assessments of traditional risk factors and disease-specific features could benefit pSS patients. Structured CV diagnostic protocols and non-invasive methods (e.g., ultrasound of the carotid artery) should be considered in patients at risk, especially in patients with a high disease activity and any additionally risk factors as well.

Our study is limited by its single-center set up, its retrospective manner and its lack of a healthy matched control group. Specific cardiovascular risk factors for women like age at menopause, hormone replacement therapy, history of pre-eclampsia, or others were not taken into account (37). There may be selection bias by pSS patients suffering from more severe disease courses in our university outpatients pSS clinic as well as the mentioned higher proportion of male patients, both leading to a higher number of patients with CVD. Despite additional assessment of medical records, recall bias in the standardized protocol may have occurred. On the other hand, we present here a very large cohort of patients with pSS who underwent both rheumatologic and neurologic standardized detailed examination. Neurologic examination did only include cerebral magnetic resonance imaging in symptomatic patients, possible asymptomatic functional changes in the brain were not assessed (46). Patients fulfill classification criteria of the time of diagnoses, therefore the cohort is more heterogenic.

In summary, we were able to identify a clinical phenotype presenting traditional cRF and clinical features of pSS that may help clinicians identify patients at risk. After diagnosis of pSS, all patients should be screened for risk factors such as hypertension and receive appropriate therapy to prevent or at least reduce sequelae such as infarction. However, further longitudinal investigations with longer follow up and larger cohorts are warranted to establish valid risk stratifications and thus improving prevention and management of CVDs in pSS patients.

## Data availability statement

The original contributions presented in the study are included in the article/Supplementary material, further inquiries can be directed to the corresponding author.

## Ethics statement

The studies involving human participants were reviewed and approved by Institutional Review Board at Hannover Medical School 8179_BO_S_2018. The patients/participants provided their written informed consent to participate in this study.

## Author contributions

CZ: data collection, writing of the manuscript, database work, and patient flow. SB: analyses, writing of the manuscript, and database work. EK, SH, and TSe: data collection and critical review. TSk, AJ, FK, KS, and TW: critical review. DE: study design, organization, analyses, and writing of the manuscript. All authors contributed to the article and approved the submitted version.
