# Supplementary material for: Premature stroke and cardiovascular risk in primary Sjögren's syndrome
Source: Front Cardiovasc Med. 2022 Dec 14;9:1048684. doi: 10.3389/fcvm.2022.1048684 (PMC9794609; doi:10.3389/fcvm.2022.1048684)
Supplement: Supplementary file 1 [file Table_1.DOCX]

**Supplementary Data**

**Premature stroke and cardiovascular risk in primary Sjögren`s Syndrome**

Clara L. Zippel^1^*, Sonja Beider^1^*, Emelie Kramer^1^, Franz Felix Konen^2^, Tabea Seeliger^2^, Thomas Skripuletz^2^, Stefanie Hirsch^1^, Alexandra Jablonka^1^, Torsten Witte^1^, Kristina Sonnenschein^3^*, Diana Ernst^1^*

^*shared authorship^

|  | **Index Patients**  **(n=28)** | **Controls**  **(n=284)** | **P - value** |
| --- | --- | --- | --- |
| Age, mean ± SD  median (min – max) | 64.1 ± 12.4  62 (36 – 85) | 57.7 ± 14.1  59 (21 – 87) | **0.040** |
| Gender (male) | 10 (35.7%) | 51 (18.0%) | **0.024** |
| Disease duration | 5.3 ± 6.3 | 4.9 ± 6.6 | 0.556 |
| Hypertension | 22 (78.6%) | 119 (41.9%) | **<0.001** |
| Hypercholesteraemia | 18 (64.3%) | 76 (26.8%) | **<0.001** |
| Diabetes mellitus | 7 (25.0%) | 35 (12.3%) | 0.061 |
| Smoking | 18 (64.3%) | 135 (47.5%) | 0.082 |
| BMI, mean ± SD | 27.7 ± 5.6 | 26.8 ± 5.8 | 0.375 |
| Physical activity | 13 (46.4%) | 169 (59.5%) | 0.346 |
| Family history | 15 (53.6%) | 119 (41.9%) | 0.235 |
| Thrombosis | 8 (28.6%) | 34 (12.0%) | **0.014** |
| Heart failure | 7 (25.0%) | 17 (6.0%) | **<0.001** |
| Atrial fibrillation | 4 (14.3%) | 18 (6.7%) | 0.118 |
| Peripheral neuropathy | 15 (53.6%) | 92 (32.4%) | **0.025** |
| CNS involvement | 6 (21.4%) | 23 (8.1%) | **0.021** |
| Fever | 0 (0.0%) | 7 (2.5 %) | 0.402 |
| Night sweats | 8 (26.8%) | 32 (11.3%) | **0.009** |
| Weight loss | 2 (7.1%) | 16 (5.6%) | 0.745 |
| ESSDAI-Score  mean ± SD  Constitutional  Lymphadenopathy  Glandular  Articular  Cutaneous  Pulmonary  Renal  Muscular  PNS  CNS  Haematological  Biological | 13.5 ± 7.7  1.1 ± 1.5  0.6 ± 1.4  0.1 ± 0.5  0.5 ± 1.2  0.2 ± 1.1  2.3 ± 5.0  0.5 ± 2.1  0.2 ± 1.1  4.8 ± 5.4  2.1 ± 4.8  0.6 ± 1.1  0.3 ± 0.7 | 10.3 ± 9.2  0.7 ± 1.5  0.6 ± 1.6  0.2 ± 0.7  0.4 ± 1.0  0.3 ± 1.2  1.9 ± 4.1  0.2 ± 1.4  0.7 ± 2.8  3.2 ± 5.0  1.0 ± 3.5  0.8 ± 1.3  0.4 ± 0.7 | **0.021**  0.060  0.869  0.675  0.697  0.439  0.781  0.069  0.501  0.063  0.064  0.800  0.194 |
| ESSDAI  high  moderate  low | 13 (46.4%)  11 (39.3%)  4 (14.3%) | 98 (34.5%)  82 (28.9%)  104 (36.6%) | **0.039** |
| ESSPRI-Score  mean ± SD  Dryness  Fatigue  Pain | 5.3 ± 2.2  4.5 ± 2.7  6.5 ± 2.8  5.0 ± 3.1 | 4.6 ± 2.2  4.1 ± 2.8  5.4 ± 2.9  4.3 ± 3.0 | 0.093  0.380  0.061  0.261 |
| ESSPRI  High (≥10) | 19 (67.9%) | 137 (48.2%) | **0.048** |
| ***SD*** standard deviation, ***BMI*** Body-Mass-Index, ***CNS*** central nervous system, ***ESSDAI*** EULAR Sjögren's syndrome disease activity index, ***PNS*** peripheral nervous system, ***ESSPRI*** EULAR Sjogren's Syndrome Patient Reported Index, | | | |

Suppl. Table 1. Comparison between primary sjögren´s syndrome patients with and without overt ischemic events.
